# Reduced lactic acidosis risk with Imeglimin: Comparison with Metformin

**DOI:** 10.14814/phy2.15151

**Published:** 2022-03-11

**Authors:** Pierre Theurey, Guillaume Vial, Eric Fontaine, Pierre‐Axel Monternier, Pascale Fouqueray, Sébastien Bolze, David E. Moller, Sophie Hallakou‐Bozec

**Affiliations:** ^1^ Poxel SA Lyon France; ^2^ University Grenoble‐Alpes INSERM U1300, Hypoxia and PhysioPathology (HP2) Laboratory Grenoble France; ^3^ Laboratory of Fundamental and Applied Bioenergetics INSERM U1055 Grenoble University Grenoble France

**Keywords:** complex I, Imeglimin, lactic acidosis, Metformin, mGPDH

## Abstract

The global prevalence of type 2 diabetes (T2D) is expected to exceed 642 million people by 2040. Metformin is a widely used biguanide T2D therapy, associated with rare but serious events of lactic acidosis, in particular with predisposing conditions (e.g., renal failure or major surgery). Imeglimin, a recently approved drug, is the first in a new class (novel mode of action) of T2D medicines. Although not a biguanide, Imeglimin shares a chemical moiety with Metformin and also modulates mitochondrial complex I activity, a potential mechanism for Metformin‐mediated lactate accumulation. We interrogated the potential for Imeglimin to induce lacticacidosis in relevant animal models and further assessed differences in key mechanisms known for Metformin's effects. In a dog model of major surgery, Metformin or Imeglimin (30–1000 mg/kg) was acutely administered, only Metformin‐induced lactate accumulation and pH decrease leading to lactic acidosis with fatality at the highest dose. Rats with gentamycin‐induced renal insufficiency received Metformin or Imeglimin (50–100 mg/kg/h), only Metformin increased lactatemia and H^+^ concentrations with mortality at higher doses. Plasma levels of Metformin and Imeglimin were similar in both models. Mice were chronically treated with Metformin or Imeglimin 200 mg/kg bid. Only Metformin produced hyperlactatemia after acute intraperitoneal glucose loading. Ex vivo measurements revealed higher mitochondrial complex I inhibition with Metformin versus slight effects with Imeglimin. Another mechanism implicated in Metformin's effects on lactate production was assessed: in isolated rat, liver mitochondria exposed to Imeglimin or Metformin, only Metformin (50–250 µM) inhibited the mitochondrial glycerol‐3‐phosphate dehydrogenase (mGPDH). In liver samples from chronically treated mice, measured mGPDH activity was lower with Metformin versus Imeglimin. These data indicate that the risk of lactic acidosis with Imeglimin treatment may be lower than with Metformin and confirm that the underlying mechanisms of action are distinct, supporting its potential utility for patients with predisposing conditions.

## Graphical abstract

Imeglimin is a recently launched medicine for type 2 diabetes (2021). Data suggest that the chemical structure and/or some aspects of mode of action may overlap with Metformin. Lactic acidosis risk with Imeglimin, a key concern for Metformin, is thus an important unanswered question. Here, we show that the risk of lactate accumulation and acidosis with Imeglimin treatment is significantly lower than with Metformin, and we confirm that the underlying mechanisms of action are distinct.

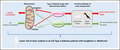

## 1 INTRODUCTION

Type 2 diabetes mellitus (T2D) is a major metabolic disease exerting tremendous pressure on public health globally, with important comorbidities and complications, including cardiovascular and renal disease (Abdul‐Ghani et al., 2017), higher risk of neurologic (Rhee et al., 2020) and mental health disorders (Ducat et al., 2014), cancer (Ling et al., 2020), and higher vulnerability to infectious diseases—even including COVID‐19 (Klonoff et al., 2021). In 2015, over 374 million patients suffered from T2D worldwide (Zheng et al., 2018), a number projected to rise to 642 million by 2040 (Zheng et al., 2018).

Metformin is a glucose‐lowering agent belonging to the biguanide family that also includes phenformin and buformin. It has been the most widely prescribed drug for T2D for several decades, and it remains the first‐in‐line treatment option recommended by the clinical guidelines of the American Diabetes Association (American Diabetes Association, 2021).

Even though Metformin has been extensively used and is generally safe and well tolerated, it has been associated with an increased probability of lactic acidosis, a rare but life‐threatening adverse effect. This occurs more frequently in association with certain risk factors such as interactions with other drugs, renal impairment, and aging (EMA, 2016; EMC, 2020; FDA, 2017). For this reason, Metformin is not recommended for initiation in patients with moderate renal insufficiency and is contraindicated in patients with severe renal impairment and chronic metabolic acidosis (EMA, 2016; EMC, 2020; FDA, 2017). Other biguanides, such as phenformin and buformin, were withdrawn from the market because of a greater lactic acidosis risk (NIH, 2021a,b). Metformin has been associated with an incidence of lactic acidosis that is estimated to be approximately 10‐fold greater than observed with other glucose‐lowering agents (Eppenga et al., 2014; Li et al., 2017; Stang et al., 1999). However, the vast majority of these cases of lactic acidosis with Metformin occurred in patients with other predisposing factors such as acute kidney injury (Mariano et al., 2017) and renal insufficiency, abnormal hepatic function, congestive heart failure, dehydration, acute hemodynamic compromise or hypoxic state, etc. (DeFronzo et al., 2016).

Lactic acidosis occurs when lactate turnover is disturbed—increased production and/or reduced clearance—leading to uncontrolled lactate accumulation and a subsequent acidotic state. Lactate is produced from the conversion of pyruvate produced by glycolysis in the liver, gut, and peripheral tissues (DeFronzo et al., 2016). The liver and kidney account for 60% and 30% of lactate clearance, respectively, through oxidation by mitochondria or entry into gluconeogenesis (DeFronzo et al., 2016). The critical role of the liver as a major site of lactate metabolism can intuitively explain why hepatic impairment, such a cirrhosis, is a risk factor for lactic acidosis.

Mechanistically speaking, the inhibition of hepatic glucose production via mild inhibition of complex I within the mitochondria respiratory chain is generally regarded as the primary mode of action of Metformin (Fontaine, 2018). Other potential mechanisms have been suggested such as indirect AMPK activation, hepatic glucagon signaling disruption by reducing cAMP levels, and inhibition of mitochondrial glycerol‐3‐phosphate dehydrogenase (mGPDH—EC 1.1.5.3) activity (Foretz et al., 2014). Metformin‐induced hyperlactatemia has been proposed to depend on the inhibition of complex I, decreasing NADH‐dependent mitochondrial oxidative phosphorylation and the consumption of pyruvate, resulting in a combined increase in lactate production and reduced metabolism leading to its accumulation (Protti et al., 2010; Wang et al., 2003).

Interestingly, Schulman and colleagues linked inhibition of hepatic gluconeogenesis to lactic acidosis by showing a Metformin‐mediated modulation of cytosolic redox state through inhibition of mGPDH; the consequences of this could include prevention of glycerol entry into gluconeogenic flux, disruption of the glycerophosphate shuttle, and accumulation of cytosolic NADH which does not favor lactate conversion to pyruvate via lactate dehydrogenase (Madiraju et al., 2014; Petersen et al., 2017).

Imeglimin is a first‐in‐class new oral medicine for the treatment of T2D. Imeglimin belongs to the chemical class of tetrahydrotriazine molecules, distinct from the biguanide class. Despite a common chemical moiety, it is not classified as biguanide by the WHO; rather, it is classified as “Other Drugs Used in Diabetes” (WHO ATC Code A10B‐X15). Like Metformin, Imeglimin at high concentrations can produce partial inhibition of mitochondrial complex I and can also reduce mitochondrial‐derived reactive oxygen species (ROS) formation. However, the predominant mode of action for Imeglimin is distinct versus Metformin (or other available antihyperglycemic medicines). Imeglimin's mechanism combines a pancreatic action to amplify glucose‐stimulated insulin secretion (along with potential protection of islet β‐cell mass) with enhanced insulin action including a reduction of hepatic glucose production and improvements in peripheral insulin sensitivity (Hallakou‐Bozec, Kergoat, Fouqueray, et al., 2021; Hallakou‐Bozec, Kergoat, Moller, et al., 2021; Hallakou‐Bozec, Vial, et al., 2021; Vial et al., 2021). These effects have been shown to relate to multiple molecular mechanisms including partial and competitive inhibition of complex I, reduction of ROS formation, restoration of complex III function, and increased β‐cell ATP generation, nicotinamide phosphoribosyltransferase (NAMPT), NAD^+^, and Ca^2+^ mobilization (Detaille et al., 2016; Hallakou‐Bozec, Kergoat, Fouqueray, et al., 2021; Hallakou‐Bozec, Vial, et al., 2021; Vial et al., 2021).

After multiple Phases II and III trials in the USA, Europe, and Japan, demonstrating significant efficacy and consistent safety, including in elderly patients and those with renal impairment (Dubourg et al., 2021; Poxel, 2021a), Imeglimin was approved for the treatment of T2D in Japan in June 2021 (Poxel, 2021b).

Although Imeglimin is not a biguanide, given its similarities with Metformin in terms of molecular action (in particular complex I inhibition), its effects on acid/base balance and lactate homeostasis were investigated in the present study and compared with Metformin in models of lactic acidosis risk.

## 2 RESULTS

### 2.1 Imeglimin does not cause lactic acidosis in healthy dogs undergoing open chest surgery

Prolonged surgery is a key risk factor for lactic acidosis, as identified in the Metformin label (FDA, 2017; Waters et al., 1999). To investigate the effects of Imeglimin compared with Metformin under such conditions, arterial lactate and pH were monitored following intraduodenal administration of Imeglimin and Metformin at 30, 100, 300, and 1000 mg/kg in healthy dogs that were subjected to open chest surgery and compared with vehicle controls.

**FIGURE 1:**
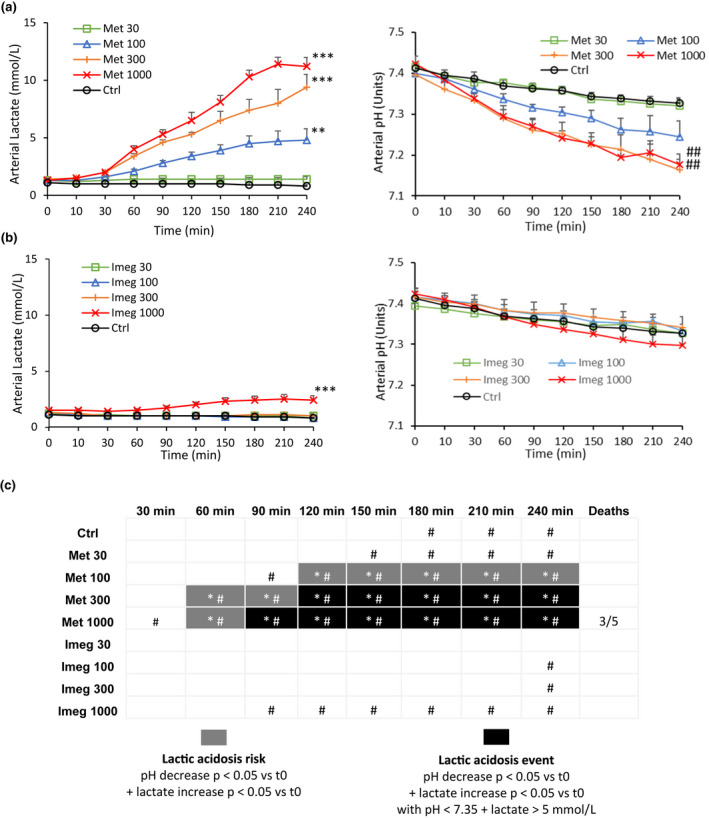
Arterial lactate and pH measured in open chest anesthetized mongrel dog. Time course of arterial lactate concentration (mmol/L) and pH (units) following intraduodenal injection of (a) Metformin or (b) Imeglimin at 30, 100, 300, or 1000 mg/kg. Values shown represent mean ± SEM of *n* = 5 animals per group. ## **p < 0.01, ***p < 0.001 versus control at t240 min (Dunnett's test) (c) Map monitoring pH decrease, lactate increase, lactic acidosis events occurrence over time for each condition, and number of deaths during the experiment. *p < 0.05 versus t0 for lactate increase (Bonferonni test). #*p* < 0.05 versus t0 for pH decrease (Bonferonni test)

In control animals injected with isotonic saline, lactate levels remained stable over the course of the experiment (Figure 1a,b left panel, Figure 1c). A tendency of progressive pH decrease of low magnitude was observed compared with baseline, significant 180 min after the administration, which may be due to the extended surgery (Figure 1a,b right panel, Figure 1c).

Intraduodenal administration of Metformin at 100, 300, and 1000 mg/kg significantly increased arterial lactate concentrations in a time‐dependent manner (Figure 1A left panel, Figure 1c). Two hundred and forty minutes after the administration, mean lactate levels were increased by 6‐, 11.8‐, and 14‐fold compared with control, respectively (Figure 1a, left panel). Arterial pH significantly decreased over time in parallel (Figure 1a, right panel, Figure 1c), to reach −0.16 and −0.15 units below mean control at 240 min after Metformin administration, for the 300 and 1000 mg/kg doses (Figure 1a, right panel); *p* values versus control for lactate concentrations and pH are displayed at the final time point in Figure 1a,b and versus baseline at every time point in Figure 1c.

Relative to vehicle controls, Imeglimin at 1000 mg/kg had a slight effect on arterial lactate but no significant effect on pH at any tested doses (Figure 1b). The plasma concentration–time curve for the two drugs, measured during the experiment, were generally similar (Figure S1).

To determine whether the observed statistically significant variations in lactate levels and pH values corresponded to standard definitions of lactic acidosis events, lactic acidosis *per se* was considered to occur with pH < 7.35 and lactate >5 mmol/L (Pang & Boysen, 2007). Metformin administration resulted in lactic acidosis occurrences after 120 and 90 min at 300 and 1000 mg/kg, respectively (Figure 1c). No lactic acidosis events occurred with Imeglimin at any tested dose (Figure 1c).

Importantly, duodenal administration of Metformin at 1000 mg/kg also resulted in the death of three animals during the experiment, while no fatal event was observed with Imeglimin at any tested dose (Figure 1c).

These results indicate that contrary to Metformin, Imeglimin does not induce lactic acidosis in dogs undergoing prolonged surgery, even at high dose, up to 1000 mg/kg.

### 2.2 Imeglimin does not cause lactic acidosis in a rat model of acute renal failure

Given the role of the kidney in lactate and Metformin clearance, renal insufficiency can lead to both plasma lactate and Metformin accumulation, thus impaired renal function is a recognized risk factor for the development of Metformin‐induced lactic acidosis (Bellomo, 2002; Rocha et al., 2013). Acute renal failure can be induced in rats by gentamicin injection (Rivas‐Cabanero et al., 1993), which directly produces tubular cell necrosis and may also cause a fall in renal blood flow. We investigated the occurrence of lactic acidosis after treatment with Imeglimin, in comparison with Metformin, in rats with gentamicin‐induced renal dysfunction. Plasma lactate and H^+^ levels were measured in parallel following the initiation of intravenous infusions of Imeglimin or Metformin at 25, 50, 75, or 100 mg/kg/h (8 ml/h/kg during 180 min) and compared with control. Blood pH values were calculated based on H^+^ concentration with pH = −log[H^+^].

**FIGURE 2:**
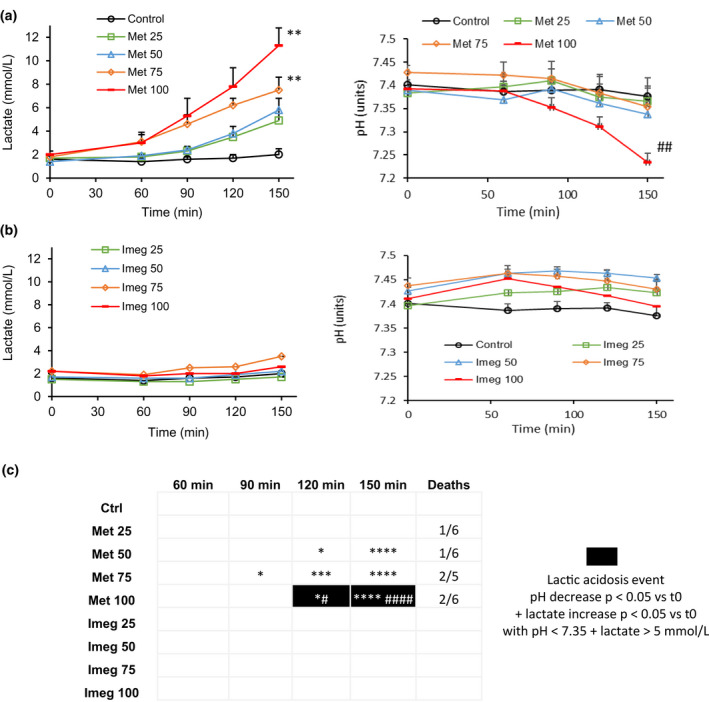
Plasma lactate and calculated pH in Sprague Dawley rats with gentamicin‐induced severe renal failure (creatinine > 2 mg/dl). Time course of plasma lactate concentration (mmol/L) and calculated pH from H+ plasma concentration (units) during infusion of (a) Metformin or (b) Imeglimin at 25, 50, 75, or 100 mg/Kg/h. Values shown represent mean ± SEM of *n* = 5–7 animals per group. ** ## *p* < 0.01 versus control at t150 min (Dunnett's test). (c) Map monitoring pH decrease, lactate increase, lactic acidosis events occurrence over time for each condition, and number of deaths during the experiment. *p < 0.05, ***p < 0.001, ****p < 0.0001 versus t0 for lactate increase (Bonferonni test). # *p* < 0.05, #### *p* < 0.0001 versus t0 for pH decrease (Bonferonni test)

In rats infused with saline (control), no significant variations in lactate or calculated pH were measured during the experiment (Figure 2).

Metformin induced time‐dependent and significant increases in lactate concentration at 50, 75, and 100 mg/kg/h (Figure 2a, left panel, Figure 2c) in rats with severe renal impairment (creatinine > 2 mg/dl). One hundred and fifty minutes after the beginning of the infusion, lactatemia was significantly increased by 3.8‐fold at 75 and 5.7‐fold at 100 mg/kg/h, compared with control (Figure 2a, left panel). In parallel, based on H^+^ concentrations, calculated pH significantly decreased over time following infusion of Metformin at 100 mg/kg/h (Figure 2a, right panel, Figure 2c). One hundred and fifty minutes after initiation of infusion, the pH level dropped by −0.14 units compared with control (Figure 2a, right panel); *p* values versus control for lactate concentrations and pH are displayed at the final time point in Figure 2a,b and versus baseline at every time point in Figure 2c.

Imeglimin had no effect on lactate or calculated pH at any tested concentration in rats with severe renal impairment (Figure 2).

To determine the conditions for which lactic acidosis arose along with significant variations in plasma lactate concentration and pH, lactic acidosis was considered to occur when pH < 7.35 and lactate >5 mmol/L (Pang & Boysen, 2007). Lactic acidosis was observed starting from 120 min for Metformin 100 mg/kg/h (Figure 2c).

Similar differences between Imeglimin and Metformin with regard to lactate and pH profiles were obtained in rats with mild (0.8 < creatinine < 1.99 mg/dl) renal failure compared with the observations in rats with severe renal failure (Figure S2).

Importantly, infusion of Metformin in rats with mild renal dysfunction at 75 and 100 mg/kg/h resulted in the death of three and five animals, respectively, and of 1, 1, 2, and 2 in rats with severe renal impairment at 25, 50, 75, and 100 mg/kg/h, respectively. No death was observed with Imeglimin at any tested dose level (Figure 2c).

Similar plasma concentrations of Metformin and Imeglimin were measured in the rats with mild and severe renal dysfunction at the end of the experiment for each dose level (Figure S3).

Taken together, these results indicate that in contrast to Metformin, Imeglimin does not induce lactic acidosis in rats with gentamicin‐induced renal failure.

### 2.3 Imeglimin displays less inhibition of complex I compared with Metformin and no effect on mGPDH activity

Given the contrasting effects of Metformin and Imeglimin on lactate levels and pH in dog and rat models at high risk of lactic acidosis, we investigated the effects of both drugs on the two main molecular pathways that have been proposed for lactate accumulation: (a) inhibition of mitochondrial respiratory chain complex I needed for consumption of lactate by the mitochondria and (b) inhibition of mGPDH, which would reduce the consumption of lactate for gluconeogenesis (Madiraju et al., 2014; Wang et al., 2003).

**FIGURE 3:**
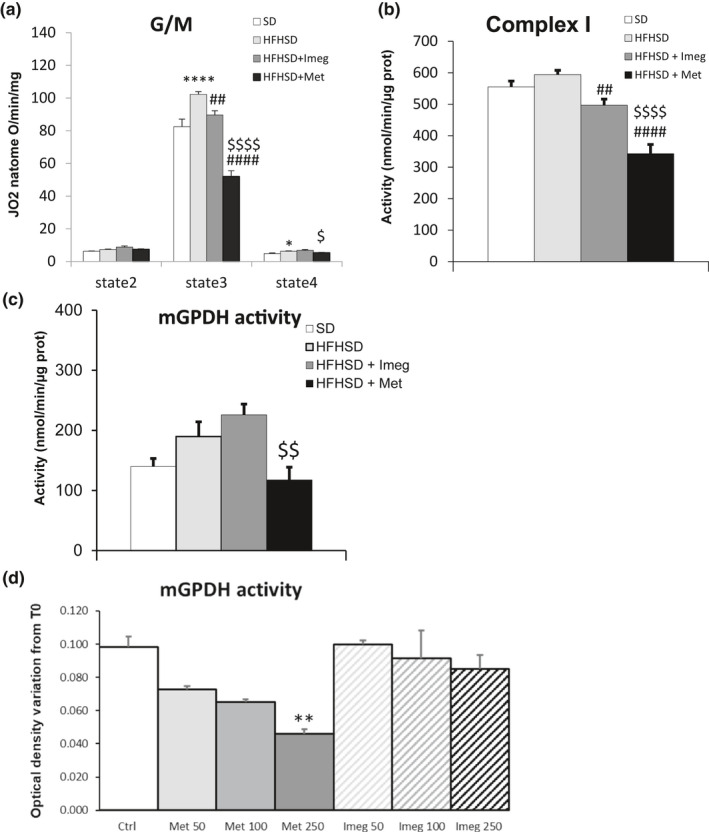
Effect of Imeglimin and Metformin treatment on complex I and mGPDH activity. (a) Oxygen consumption of liver mitochondria isolated from HFHSD mice following 6 weeks of oral gavage with Metformin or Imeglimin at 200 mg/kg bid, by incubating mitochondria (1 mg/ml) with glutamate (5 mmol/L)/malate (2.5 mmol/L) without ADP (state 2), in the presence of ADP (1 mmol/L, state 3) or oligomycin (5 μg/ml, state 4). *n* = 8–13 animals per group.*p < 0.05, ****p < 0.0001 versus SD; ## *p* < 0.01, #### *p* < 0.0001 versus HFD; $ *p* < 0.05, $$$$ *p* < 0.0001 versus HFHSD + Imeg (Tukey's test). (b) Rotenone‐sensitive NADH‐ubiquinone oxidoreductase, and (c) mGPDH activity in liver mitochondria isolated from HFHSD mice following 6 weeks of oral gavage with Metformin or Imeglimin at 200 mg/kg bid, using a spectrofluorometer and specific substrates. *n* = 9–14 animals per group. ## *p* < 0.01, #### *p* < 0.0001 versus HFHSD; $$ *p* < 0.01, $$$$ *p* < 0.0001 versus HFHSD + Met (Tukey's test). (d) mGPDH activity from isolated rat liver mitochondria measured by gain of absorbance at 600 nm in KCl buffer, performed using DCIP 1 mmol/L and FAD(H2) 100 µmol/L as an electron donor, GP 10 mmol/L as a substrate, and 130 µg of total mitochondrial as enzyme, following 10 min incubation with Metformin or Imeglimin at 50, 100, and 250 µmol/L. *n* = 3–6 per condition. ** *p* < 0.01 versus ctrl (Dunn's test). All histograms present mean ± SEM

Effects on complex I activity were assessed by measuring glutamate‐/malate‐dependent respiration and by enzymatic assay in isolated liver mitochondria from mice with T2D (induced via high‐fat high‐sucrose diet, HFHSD) treated by oral dosing with 200 mg/kg bid for 6 weeks with Metformin or Imeglimin. As we previously described (Vial et al., 2015), complex I‐dependent respiration was significantly increased in HFHSD mice compared with control standard diet (SD) mice in the state 3 of respiration (with 1 mmol/L ADP, +24% vs. control, Figure 3a). Interestingly, Imeglimin treatment in HFHSD mice slightly but significantly reduced oxygen consumption compared with control HFHSD condition—down to a level close to SD (−12.4% vs. HFHSD, Figure 3a). In contrast, Metformin inhibited respiration to a significantly higher extent compared with Imeglimin (−49.2% vs. HFHSD, −42% vs. HFHSD + Imeglimin, Figure 3a). Of note, respiration after oligomycin addition (5 μg/ml, state 4) was higher in HFHSD versus SD (+29%), and with Imeglimin treatment compared with Metformin (+21%, Figure 3a) suggesting variations in mitochondrial coupling. Importantly, plasma lactate concentrations measured at 5 weeks of treatment after intraperitoneal injection of 1.5 g/kg glucose (to stimulate glycolysis) were significantly increased by Metformin (+67%, Table 1), while Imeglimin did not show such an effect (Table 1).

**TABLE 1:** Plasma lactate concentration in HFHSD mice following 5 weeks of oral gavage with Metformin or Imeglimin at 200 mg/kg bid, in a 6‐h fasted state and 2‐h postdose. Blood samples were collected 60 min after i.p. injection of glucose (1.5 g/kg). *n* = 10–14 animals/group

Lactate concentration mmol/L	SD (Control)	HFHSD	HFHSD + Imeg	HFHSD + Met
Mean	7.5	10.5	14.0	17.6
SEM	0.3	0.8	1.2	1.3
	*p* value Tukey's test	ns versus SD	ns versus HFHSD	*p* < 0.001 versus HFHSD

To complement the respiration experiments, we used an enzymatic assay to monitor complex I activity by measuring NADH oxidation with spectrophotometry. Contrary to respiration measurements, no increase in complex I activity was measured in HFHSD mice compared with SD. However, in accord with oxygen consumption measurements, Imeglimin treatment in HFHSD mice slightly and significantly reduced complex I activity compared with control HFHSD mice (−16.4%, Figure 3b); however, this effect was substantially less prominent than that observed with Metformin (−42.3% vs. HFHSD, −31% vs. HFHSD + Imeglimin, Figure 3b).

These results indicate that the extent of Imeglimin's inhibitory effect on complex I is significantly lower than Metformin.

Finally, we assessed the effect of both drugs on mGPDH activity in the liver of HFHSD mice treated with 200 mg/kg/day Imeglimin or Metformin for 6 weeks, or in rat isolated mitochondria exposed to 50, 100, and 250 µmol/L Imeglimin or Metformin, by spectrophotometry using electron donors and glycerol‐3‐phosphate as a substrate. mGPDH activity was not significantly altered in HFHSD mice compared with SD and Imeglimin treatment had no significant effect on the enzyme's activity in mitochondria isolated from treated HFHSD mice (Figure 3c). In contrast, Metformin tended to reduce mGPDH activity compared with control HFHSD mice but without reaching significance (−38%, *p* < 0.07, Figure 3c). Interestingly, mGPDH activity upon Metformin treatment was significantly lower compared with mean values obtained with Imeglimin treatment (−47.9%, Figure 3c). To further assess these differences, in vitro experiments were performed by using isolated mitochondria from wild‐type rats that were incubated with Imeglimin or Metformin. mGPDH activity was followed over time following the addition of each drug. Ten minutes after the beginning of the drug incubation, Metformin reduced mGPDH activity at all tested concentrations but reached significance at 250 µmol/L (−53.4%, Figure 3d). Similar to the results obtained ex vivo with isolated mitochondria from treated HFHSD mice, Imeglimin had no effect on mGPDH activity in isolated rat mitochondria (Figure 3d).

These results indicate that contrary to Metformin, Imeglimin does not inhibit mGPDH activity.

## 3 DISCUSSION

Metformin remains the recommended first‐line treatment to treat type 2 diabetes in the USA and Europe. Even though widely prescribed and generally well tolerated, rare but fatal cases of lactic acidosis have been identified in patients with predisposing risk factors, in particular chronic kidney disease. For this reason, Metformin is contraindicated in patients with an eGFR below 30 ml/min/1.73 m^2^ and regulatory authorities recommend extra care (monitoring of risk factors, in particular, for certain product combinations) or avoiding initiation of treatment with less‐severe renal insufficiency—up to 59 mL/min/1.73 m^2^ (EMA, 2016; EMC, 2020; FDA, 2017), corresponding to chronic kidney disease stages 4 and 3, respectively. Interestingly, beyond lactic acidosis risk considerations, both Metformin and Imeglimin have shown protective effects on renal function (Lachaux et al., 2020; Mariano & Biancone, 2021).

Given that Metformin and Imeglimin share some common structural and mechanistic features (Hallakou‐Bozec, Vial, et al., 2021), we compared Metformin to Imeglimin in preclinical models sensitive to lactate accumulation and lactic acidosis, including open chest surgery and gentamycin‐induced renal impairment (Rivas‐Cabanero et al., 1993; Waters et al., 1999).

Following the administration of increasing doses of Imeglimin in healthy dogs subjected to open chest surgery and rats with gentamicin‐induced mild and severe renal impairment, we did not measure any occurrence of hyperlactatemia (lactate > 5 mmol/L) or lactic acidosis (lactate > 5 mmol/L + pH < 7.35) events nor physiologically relevant and significant increases in plasma lactate concentration or decreases in pH.

In contrast, administration of similar doses of Metformin in both animal models resulted in significant increases in mean plasma lactate levels and decreases in pH along with occurrences of overt lactic acidosis according to standard clinical definitions (Pang & Boysen, 2007). Although a precise cause of death was not established, it is important to note that several deaths occurred only with Metformin, in particular at higher dose levels, whereas no deaths—either related to lactic acidosis events or not—occurred with Imeglimin despite doses and plasma concentrations reached in the models are far greater those used in clinical settings (Clemence et al., 2020). Moreover, we confirmed that plasma exposure levels to both drugs were similar in the two models. Accordingly, Metformin and Imeglimin have overlapping plasma exposure in humans at their respective therapeutic doses (Clemence et al., 2020).

Imeglimin's mode of action involves dual effects to amplify glucose‐stimulated insulin secretion and to enhance insulin action in peripheral tissues such as skeletal muscle (Hallakou‐Bozec, Vial, et al., 2021). The former effect involves a novel pathway in pancreatic β‐cells (Hallakou‐Bozec, Kergoat, Fouqueray, et al., 2021) and has been shown to translate to humans in a clinical trial context (Pacini et al., 2015). On the other hand, there are no published data implicating a similar effect of Metformin. Despite these clear distinctions, there is some overlap in the mode of action for Metformin and Imeglimin since both have been shown to suppress gluconeogenesis via partial mitochondrial complex I inhibition (Vial et al., 2021). Complex I inhibition has been suggested as a potential mechanism linking Metformin to lactic acidosis, by reducing pyruvate consumption leading to lactate accumulation (Protti et al., 2010; Wang et al., 2003). However, previous studies have shown that Metformin mediates uncompetitive inhibition of complex I, whereas Imeglimin's effect on complex I is competitive (Vial et al., 2021). Thus, Imeglimin reduces the affinity of NADH for the respiratory chain without affecting the oxygen consumption rate in intact rat hepatocytes. In addition, no net effect of Imeglimin to suppress mitochondrial oxidative respiration was shown in cultured human cells (Detaille et al., 2016). Therefore, it has been previously suggested that Imeglimin may be associated with a lower risk of lactic acidosis versus Metformin (Vial et al., 2021). These important differences may account, at least in part, for the lack of Imeglimin‐induced lactic acidosis that we observed, compared with Metformin in the present study.

In the context of chronic treatment using HFHSD mice, we did detect a modest but significant ex vivo inhibitory effect of Imeglimin on complex I‐dependent (glutamate/malate) respiration in isolated mitochondria; however, the effect was almost 4‐fold greater with Metformin than with Imeglimin compared with control HFHSD. This difference—in the degree of effect on complex I—generally aligns with the aforementioned published results and further suggests that Imeglimin may be associated with reduced risk of lactic acidosis.

Beyond lactate consumption by mitochondria through pyruvate, gluconeogenesis provides another metabolic pathway for lactate consumption that would act to prevent its accumulation; this helps to explain why gluconeogenic organs, liver and kidney, are major contributors in the control of lactate levels and systemic acid/base balance (DeFronzo et al., 2016). Although both Metformin and Imeglimin share an ability to inhibit gluconeogenesis, higher concentrations of Imeglimin are required to produce this effect in isolated rat hepatocytes versus Metformin (Vial et al., 2021). An important potential aspect of Metformin's mode of action was unveiled in studies showing that it functions to also inhibit mGPDH; this results in reduced entry of glycerol into gluconeogenic flux, disrupting the glycerophosphate shuttle and leading to accumulation of cytosolic NADH which favors lactate accumulation (Madiraju et al., 2014; Petersen et al., 2017). Even though this mechanism was challenged in contradictory studies and is still a matter of debate (MacDonald et al., 2021), our results are in line with this hypothesis in isolated mitochondria from HFHSD mice treated with Metformin and in isolated mitochondria from wild‐type rats incubated with Metformin. Importantly, we did not observe any inhibitory effect of Imeglimin under similar conditions at any tested concentration. These results thus serve to further reinforce the concept of reduced lactic acidosis risk compared with Metformin.

In conclusion, substantial differences between the impact of Metformin and Imeglimin on the two major suggested mechanisms driving Metformin‐induced lactic acidosis, complex I and mGPDH inhibition, were documented. More importantly, the lack of observed effects of Imeglimin on lactate levels, blood pH, lactic acidosis occurrence, and fatal events seen in high‐risk preclinical models, support the notion that Imeglimin is a distinct medicine with a different mode of action and that it may be associated with an intrinsically lower risk of lactic acidosis compared with Metformin, in particular in patients with predisposing conditions. To date, no cases of lactic acidosis or meaningful differences in mean lactate levels compared with placebo were measured in patients receiving Imeglimin, including studies testing its effects as an add‐on therapy to Metformin. The Imeglimin development program consisted of nine Phase I studies, five Phase II studies, and three Phase III studies (these studies did not include CKD3b and four patients), for a total of 2301 subjects exposed to Imeglimin (Dubourg et al., 2021). Metformin remains a crucial drug for diabetes care with a highly favorable cost–benefit ratio. However, given these observations and the persistent nature of unmet medical needs in higher risk populations of patients with T2D, such as the elderly or those with renal impairment, further studies to define the efficacy and safety tolerability of Imeglimin in such patients appears to be warranted.

## 4 MATERIALS AND METHODS

### 4.1 Compounds

#### 4.1.1 Rat renal failure and open chest dog

Imeglimin and Metformin were chemically synthesized by Merck Santé.

#### 4.1.2 mGPDH‐isolated mitochondria

Imeglimin, Poxel, batch number K37745124. Metformin chemically synthesized by Merck Santé.

### 4.2 Open chest surgery dog model

Male and female healthy mongrel dogs were handled according to the guidelines of the American Physiological Society. The bioethical committee of the district of Darmstadt, Germany approved the protocol.

Anesthesia was initiated with 15 mg/kg BW thiopental i.v. (V. cephalica) and maintained by ventilating with a gas mixture containing oxygen and dinitrogen monoxide (ratio 1:2) and 0.3% isoflurane. Additionally, piritramide (narcotic painkiller) was continuously infused i.v. at a dose of 0.25 mg/kg BW/h. During the experiment, the animals received pancuronium bromide (4.0 mg/animal i.v.) for muscle relaxation if indicated. Open chest surgery was performed as described in supplementary materials. Arterial and venous blood samples (2 ml) were drawn into blood sampling syringes Pico 50 (Radiometer) for subsequent analysis with an Analyzer (Radiometer 620, Radiometer). Analytical samples were drawn into syringe containers with already added EDTA (Monovette Sarstedt) and spun 30 min at 3000 rpm at 0°C. The plasma phase was pipetted into cups (Eppendorf) and immediately stored at −70°C. After completion of the experiments, the samples were sent on dry ice to the Institute for Drug Metabolism and Pharmacokinetic in Grafing, Germany, for subsequent analysis.

The control measurement was taken at time 0. Subsequently, the animals received the study substance at time 0 min over 1 min intraduodenally. Measurements were taken every 5 min during the first 30 min and every 15 min in the following 60 min. Thereafter, until the end of the observation period at 240 min, every 30 min measurements were taken. At time points 0, 10, 30, 45, 60, 90, 120, 180, 210, and 240 min, acid/base balance, blood gases, and metabolic parameters were measured.

At the end of the observation period, the animals were sacrificed by induction of ventricular fibrillation.

### 4.3 Rat model of renal failure

Male Sprague Dawley rats (Charles River–L'Arbresle—France), handled as described in supplementary materials, were administered gentamicin sulfate from SIGMA (Ref. G3632) dissolved in isotonic saline solution (sodium chloride 9/1000) at 200 mg/ml concentration, by subcutaneous route (administration volume: 1 ml/kg weight) once a day during 4 days. The creatinine level, correlated with the degree of the renal function failure, was tested for each rat 7 days after the first injection of Gentamicin. The creatinine level was determined on the Monarch Chemistry Systems using the IL Test creatinine (Ref. 181672–00) in rat plasma samples. Animals were randomized into two groups: mild renal failure with 0.8 < creatinine <1.99 mg/dl and severe renal failure with creatinine >2 mg/dl. The animals were catheterized for the study. The rats were anesthetized with intraperitoneal sodium pentobarbital (50 mg/kg). Catheters made from medical grade silicone were implanted into right jugular vein and filled with heparin solution in saline (125 UI/ml). The catheters were exteriorized at the nape of the neck and anchored to the skin. Two days after the surgery, the animals were randomly assigned to control treatment groups. Drug infusion was performed using a basic syringe pump (Harvard Apparatus Inc.). Imeglimin or Metformin dissolved in saline were administered at a constant rate of 8 ml/h/kg for 180 min. Controls were infused with saline at the same constant rate. The infusion doses were 25, 50, 75, and 100 mg/h/kg for both Imeglimin and Metformin.

Blood samples used for the determination of the different parameters were drawn from the tail vein at 0, 60, 90, 120, 150, and 180 min. Using a Radiometer System ABL 615 (Radiometer), lactate and H^+^ concentrations were determined. At the end of infusion period, blood was taken for the determination of plasma drug concentrations.

### 4.4 Lactate levels, complex I, and mGPDH activities in high‐fat high‐sucrose diet mice

Male C57BL/6JOlaHsd mice (Harlan) at 4 weeks of age were used. Animal procedures were conducted in accordance with the institutional guidelines for the care and use of laboratory animals. At 5–6 weeks of age, mice were divided into two groups: 10 mice with free access to a standard chow diet (SD, 57% carbohydrate, 5% fat, and 18% protein; A04 Safe) and 40 mice with free access to a pelleted HFHS (36% fat, 35% carbohydrate [50% sucrose], and 19.8% protein; 260HF Safe). Each of these diets were consumed for 16 weeks. Animals were treated with Imeglimin or Metformin at 200 mg/kg bid in Methylcellulose 0.5% by oral gavage during the 6 last weeks of HFHS diet feeding period. SD and HFHSD control mice were treated by oral gavage with only Methylcellulose 0.5%, used as vehicle for drug treatments.

Lactate levels were measured after 5 weeks of treatment (week 15 of diet) in a 6‐h fasted state and 2‐h postgavage. Before and after 60‐min glucose injection (1.5 g/kg), plasma lactate was measured by spectrophotometric assay as described in supplementary material.

For complex I respiration/activity and mGPDH activity, mice were anesthetized using isoflurane at the end of the protocol; then animals were sacrificed by cervical dislocation, the liver was rapidly excised and weighed. Mouse liver mitochondria were isolated according to a standard differential centrifugation procedure in 250 mM sucrose, 20 mM Tris–HCl, 1 mM EGTA, pH 7.4. Protein concentrations were determined using the bicinchoninic acid assay using BSA as a standard (Pierce). The rate of mitochondrial oxygen consumption (JO_2_) was measured at 30°C using a Clark‐type O_2_ electrode in a 1 ml chamber filled with respiration buffer: 125 mmol/L KCl, 10 mmol/L Pi‐Tris, 20 mmol/L Tris–HCl, 0.1 mmol/L EGTA, pH 7.2, and using 1 mg of mitochondrial proteins/ml. Measurements were carried out in the presence of either glutamate (5 mmol/L)/malate (2.5 mmo/L) and/or succinate (5 mmol/L) as substrates, after the addition of 1 mmol/L ADP (state 3), followed by the addition of 0.25 mg/ml oligomycin (state 4).

Rotenone‐sensitive NADH‐ubiquinone oxidoreductase (EC 1.6.5.3, complex I) was assayed using 100 µmol/L decylubiquinone as an electron acceptor and 200 µmol/L NADH as a donor, in a 10 mmol/L KH_2_PO_4_/K_2_HPO_4_ buffer, pH 7.5 containing 3.75 mg/ml BSA, 2 mmol/L KCN 7.5 µmol/L antimycin A. Oxidation of NADH was then measured at 340 nm, before and after the addition of 4 µmol/L rotenone to allow the calculation of the rotenone‐sensitive‐specific activity which is a characteristic of complex I (Vial et al., 2015).

Mitochondrial mGPDH (EC 1.1.5.3) activity was measured in isolated mitochondria after three cycles of freezing and thawing, by measuring the reduction of 50 µmol/L dichloro‐indophenol at 600 nm by mGPDH. Measurements were performed at 37°C with decylubiquinone 50 µmol/L and sn‐glycerol‐3‐phosphate in a 50 mmol/L KH_2_PO_4_/K_2_HPO_4_ buffer, pH 7.5 containing 2.5 mg/ml BSA, 9.3 µmol/L antimycin A, and 5 µmol/L rotenone (Vial et al., 2011).

### 4.5 mGPDH in isolated rat mitochondria

Mitochondria from Wistar rat livers were commercially provided from Xenotech and mitochondria were lysed in Tris/Sucrose/EDTA/DTT/Triton buffer. The protein concentration within mitochondria was provided by Xenotech.

Measurement of mGPDH activity from rat liver mitochondria was performed using DCIP (from Fluka 36180‐56‐F) as an electron acceptor, FAD(H2) as an electron donor (from Sigma F6625), GP as a substrate for the enzyme (from Sigma 61668); gain of absorbance was determined at 600 nm in KCl buffer (50 mmol/L KCl, 1 mmol/L Tris–HCl, 1 mmol/L EDTA, 1 mg/ml BSA, 1 mmol/L KCN, and pH 7.4). Enzyme activity curves were expressed as a Delta optic density between time 0 and different post exposures times to determine kinetics.

### 4.6 Statistics

For in vivo experiments following plasma lactate and pH over time in rats and dogs, one‐way ANOVA was applied followed by Bonferonni multiple comparison test to compare versus t0 and Dunnett's test to compare versus control. For the study of the effects of Imeglimin and Metformin treatment on complex I and mGPDH activity, a one‐way ANOVA followed by Tukey' test was applied for the experiments in HFHSD mice and a Kruskall–Wallis followed by a Dunn's test using measures within the linear portion of enzyme curve activity was used for the incubation of isolated mitochondria with the drugs. Results were considered statistically significant at *p* < 0.05.

## CONFLICT OF INTEREST

PT, PAM, PF, SB, DM, and SHB are employees of Poxel SA. GV and EF received fees for research activities from Poxel SA.

## AUTHOR CONTRIBUTION

Writing of the manuscript: all authors. Studies design: GV, EF, PF, SB, and SHB. Data analysis and interpretation: all authors.

## Supporting information



Fig S1
