# Supplementary material for: Reduced lactic acidosis risk with Imeglimin: Comparison with Metformin
Source: Physiol Rep. 2022 Mar 11;10(5):e15151. doi: 10.14814/phy2.15151 (PMC8915386; doi:10.14814/phy2.15151)
Supplement: Supplementary file 1 — Fig S1 [file PHY2-10-e15151-s001.pptx]

## Slide 1
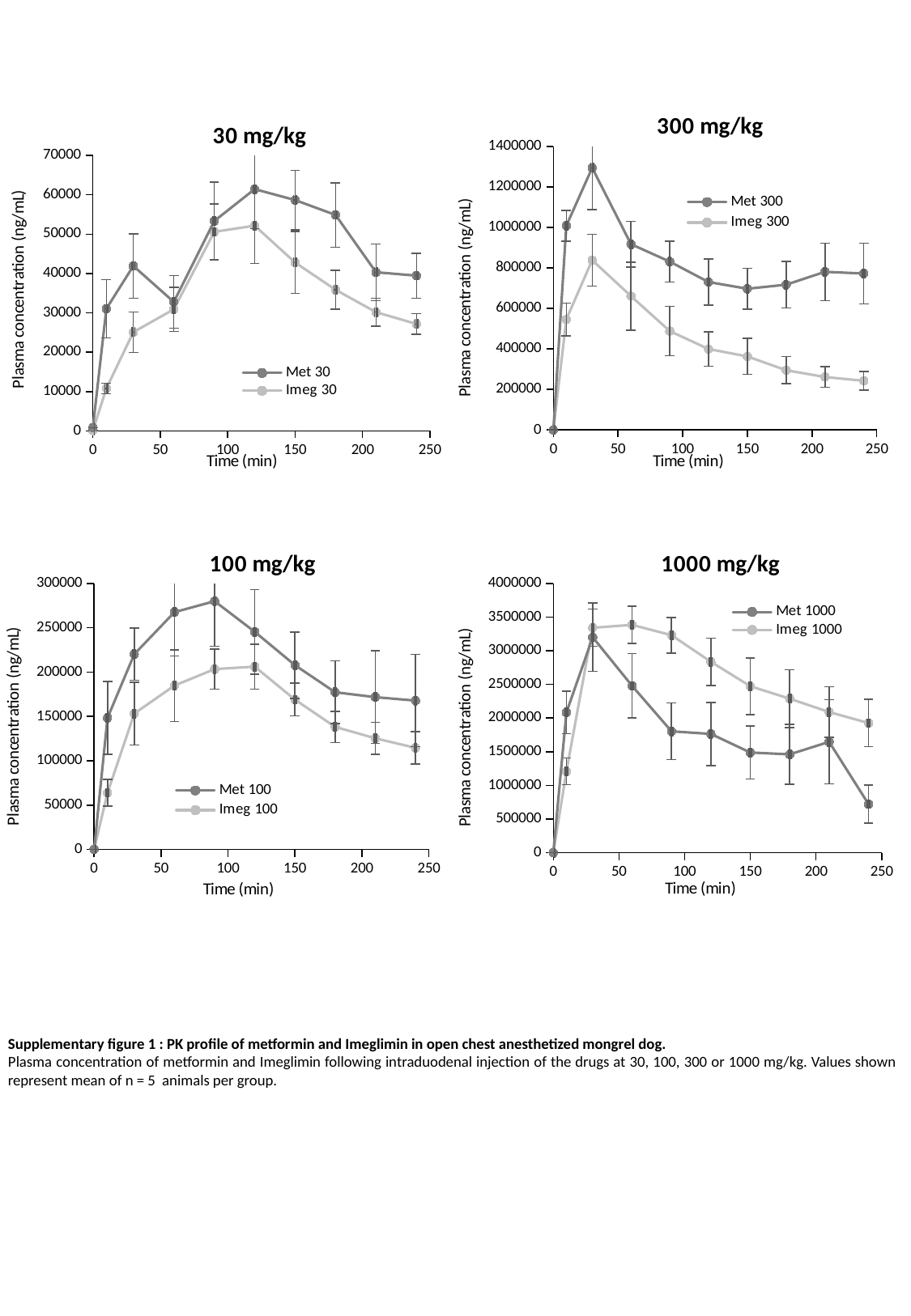

### Chart: 300 mg/kg
| Category | Met 300 | Imeg 300 |
|---|---|---|
### Chart: 30 mg/kg
| Category | Met 30 | Imeg 30 |
|---|---|---|
### Chart: 100 mg/kg
| Category | Met 100 | Imeg 100 |
|---|---|---|
### Chart: 1000 mg/kg
| Category | Met 1000 | Imeg 1000 |
|---|---|---|Supplementary figure 1 : PK profile of metformin and Imeglimin in open chest anesthetized mongrel dog.
Plasma concentration of metformin and Imeglimin following intraduodenal injection of the drugs at 30, 100, 300 or 1000 mg/kg. Values shown represent mean of n = 5 animals per group.

## Slide 2
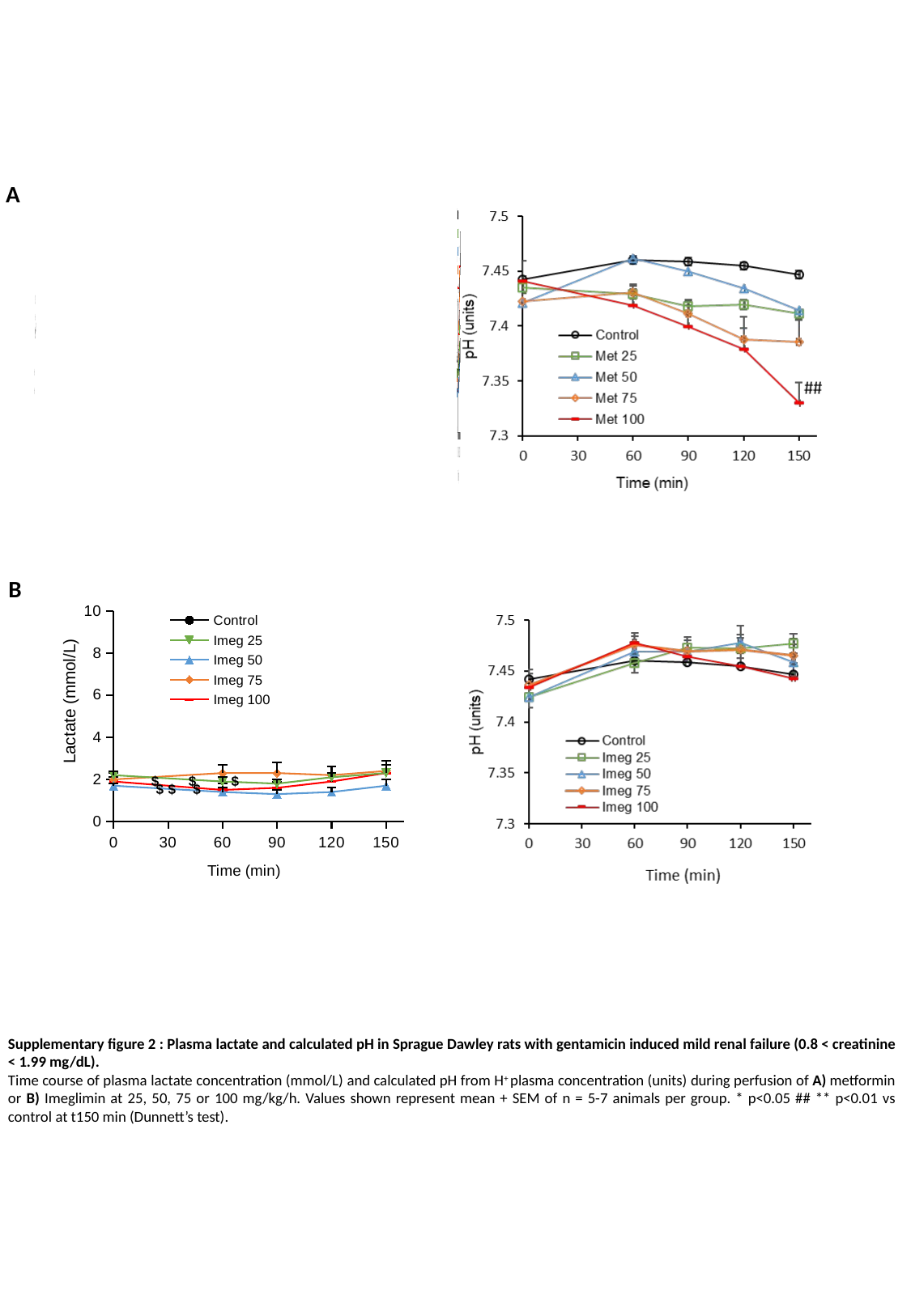

A
### Chart
| Category | Control | Met 25 | Met 50 | Met 75 | Met 100 |
|---|---|---|---|---|---|
B
### Chart
| Category | Control | Imeg 25 | Imeg 50 | Imeg 75 | Imeg 100 |
|---|---|---|---|---|---|
Supplementary figure 2 : Plasma lactate and calculated pH in Sprague Dawley rats with gentamicin induced mild renal failure (0.8 < creatinine < 1.99 mg/dL).
Time course of plasma lactate concentration (mmol/L) and calculated pH from H+ plasma concentration (units) during perfusion of A) metformin or B) Imeglimin at 25, 50, 75 or 100 mg/kg/h. Values shown represent mean + SEM of n = 5-7 animals per group. * p<0.05 ## ** p<0.01 vs control at t150 min (Dunnett’s test).

## Slide 3
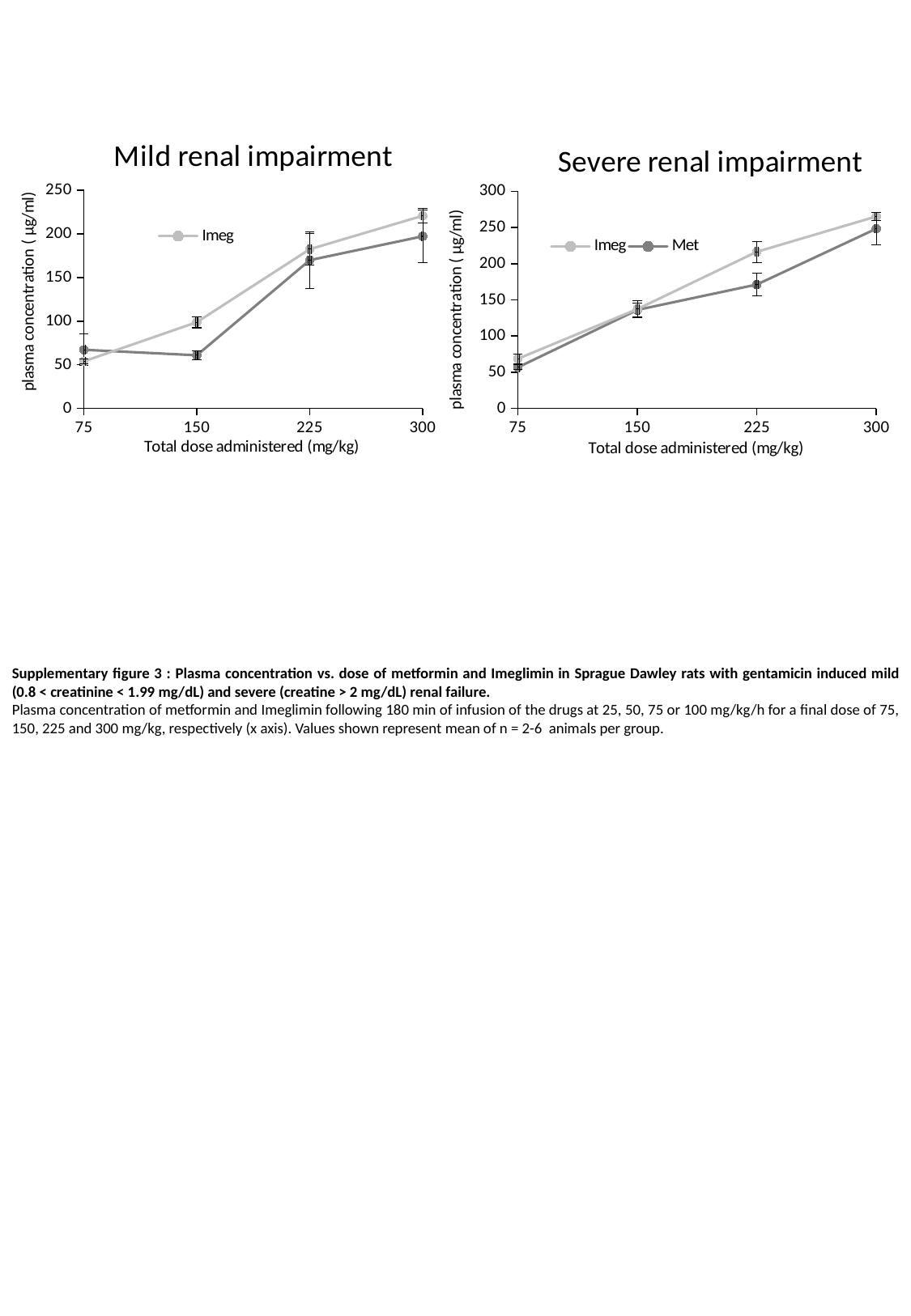

### Chart: Severe renal impairment
| Category | Imeg | Met |
|---|---|---|
### Chart: Mild renal impairment
| Category | Imeg | Met |
|---|---|---|Supplementary figure 3 : Plasma concentration vs. dose of metformin and Imeglimin in Sprague Dawley rats with gentamicin induced mild (0.8 < creatinine < 1.99 mg/dL) and severe (creatine > 2 mg/dL) renal failure.
Plasma concentration of metformin and Imeglimin following 180 min of infusion of the drugs at 25, 50, 75 or 100 mg/kg/h for a final dose of 75, 150, 225 and 300 mg/kg, respectively (x axis). Values shown represent mean of n = 2-6 animals per group.
